# Decomposition of a Multiscale Entropy Tensor for Sleep Stage Identification in Preterm Infants

**DOI:** 10.3390/e21100936

**Published:** 2019-09-25

**Authors:** Ofelie De Wel, Mario Lavanga, Alexander Caicedo, Katrien Jansen, Gunnar Naulaers, Sabine Van Huffel

**Affiliations:** 1 Department of Electrical Engineering (ESAT), STADIUS Center for Dynamical Systems, Signal Processing and Data Analytics, KU Leuven, 3001 Leuven, Belgium; mario.lavanga@kuleuven.be (M.L.); Sabine.VanHuffel@esat.kuleuven.be (S.V.H.); 2 Department of Applied Mathematics and Computer Science, Universidad del Rosario, Bogotá 111711, Colombia; alexander.caicedo@urosario.edu.co; 3 Department of Development and Regeneration, Neonatal Intensive Care Unit, University Hospitals Leuven, 3000 Leuven, Belgium; katrien.jansen@uzleuven.be (K.J.); gunnar.naulaers@uzleuven.be (G.N.); 4 Department of Development and Regeneration, Child Neurology, University Hospitals Leuven, 3000 Leuven, Belgium

**Keywords:** CPD, EEG, multiscale entropy, sleep staging, tensor decomposition, preterm neonate

## Abstract

Established sleep cycling is one of the main hallmarks of early brain development in preterm infants, therefore, automated classification of the sleep stages in preterm infants can be used to assess the neonate’s cerebral maturation. Tensor algebra is a powerful tool to analyze multidimensional data and has proven successful in many applications. In this paper, a novel unsupervised algorithm to identify neonatal sleep stages based on the decomposition of a multiscale entropy tensor is presented. The method relies on the difference in electroencephalography(EEG) complexity between the neonatal sleep stages and is evaluated on a dataset of 97 EEG recordings. An average sensitivity, specificity, accuracy and area under the receiver operating characteristic curve of 0.80, 0.79, 0.79 and 0.87 was obtained if the rank of the tensor decomposition is selected based on the age of the infant.

## 1. Introduction

In human infants, the emergence of sleep cycles occurs at approximately 26 to 28 weeks postmenstrual age (PMA) [1]. During maturation of the sleep architecture, the distribution and duration of specific sleep states change gradually. Very young preterm infants have an abundant amount of sleep, and active sleep is the dominant sleep stage. From then on, the proportion of time spent asleep decreases, while the relative amount of quiet sleep increases. Near term age, both active sleep and quiet sleep constitute approximately half of the total sleep time [2,3]. Existing research recognises the importance of sleep in early brain development [1,3,4]. Sleep and established sleep cycling play a vital role in normal neurosensory development, learning processes, memory consolidation and in the protection of the infant’s brain plasticity [1]. Moreover, studies such as that conducted by Shellhaas et al [5] have shown that the presence of sleep cycling and the quantity and quality of each sleep state are associated with neurodevelopmental outcomes [6,7,8].

Most prematurely born infants stay in the neonatal intensive care unit (NICU) during the first critical weeks of rapid growth and development of the brain. In the NICU, neonates are exposed to a myriad of noxious environmental stimuli, such as high noise and light levels and painful procedures, which might disrupt their sleep state organization. In recent years, there has been an increasing interest in strategies to promote sleep in the NICU environment (e.g., kangaroo care, massage therapy, cycle lighting, etc.) [7,9].

Real-time automated identification of behavioural states can be used to optimize the planning of NICU caregiving in order to reduce disturbance of sleep-wake cyclicity [10]. Moreover, an automated sleep staging algorithm can be used to assess the sleep architecture and by that the functional brain maturation. In view of all that has been mentioned so far, one may suppose that there is a need to assess the sleep staging of neonates in order to provide developmentally appropriate care.

A number of algorithms for sleep stage classification in preterm neonates have been developed. The majority of these approaches are supervised and combine a set of electroencephalography (EEG) features (e.g., temporal features, spectral features, spatial features, complexity features) with a classification algorithm [11,12]. Recently, deep learning has also found its way to sleep staging in preterm infants [13]. Finally, Dereymaeker et al. [14] have proposed a cluster-based algorithm for quiet sleep detection.

This paper proposes a novel unsupervised method to discriminate quiet sleep from non-quiet sleep in preterm infants. In this study, a tensor-based method exploiting the differences in EEG complexity between different vigilance states will be used. Due to the increasing amount of data being collected, and the specific properties of tensor decompositions, multiway analysis has received increasing attention during recent decades. Tensor algebra has been used in a broad range of applications, such as image and video processing, machine learning and biomedical applications [15,16]. To the best of our knowledge, this is the first paper where tensor decompositions are used to discriminate sleep stages in preterm neonates. Therefore, this paper can serve as a proof of concept and illustrate how tensor decompositions can be used in biomedical applications, and more specifically in a classification problem based on neonatal EEG.

The remaining part of the paper proceeds as follows. The first section of this paper will describe the dataset. It will then go on to the explanation of the different steps of the proposed method. Afterwards the results of the algorithm will be reported and discussed.

## 2. Database

The proposed method is evaluated on a dataset consisting of EEG signals recorded at the neonatal intensive care unit of the University Hospitals of Leuven, Belgium. All neonates included in the study were born between 2012 and 2014 at a gestational age below 32 weeks (range: 24 weeks 4 days–32 weeks). In total 97 multichannel EEG signals were measured from 26 neonates at a postmenstrual age (PMA) between 27 and 42 weeks. So, each subject had at least two serial EEG recordings during their stay in the NICU. The study was approved by the Ethics committee of the University Hospitals of Leuven and parental consent was obtained for all recruited patients. Criteria for selecting the subjects were as follows: a normal neurodevelopmental outcome at 9 and 24 months corrected age (Bayley Scales of Infant Development-II, mental and motor score > 85), no severe brain lesions assessed by ultrasound and not taking any sedative or antiepileptic drugs during the EEG registration. EEG was recorded using nine electrodes: Fp1, Fp2, C3, C4, T3, T4, O1, O2 and Cz using the modified 10–20 system [17]. Electrode Cz served as a reference and was not taken into account during the analysis. The EEG data were acquired at a sampling frequency of 250 Hz using BrainRT equipment (OSG bvba, Rumst, Belgium). The analysis of the data was carried out in Matlab 2017b (The MathWorks, Inc., Natick, MA, USA), and the tensor decompositions were performed using Tensorlab [18].

Quiet sleep periods were annotated by two independent expert clinicians upon agreement. All other sleep states are merged and will be referred to as non-quiet sleep. The goal of the proposed algorithm is to automatically label EEG segments as either quiet sleep (QS) or non-quiet sleep (NQS). The most important feature in discriminating quiet sleep from non-quiet sleep is the continuity of the EEG trace. Generally, the EEG signal is relatively more discontinuous during quiet sleep compared to non-quiet sleep. Moreover, non-quiet sleep is characterized by a higher variability of the cardiorespiratory pattern and more body movements [4].

## 3. The Proposed Tensor-Based Sleep Stage Identification Method

The pipeline of the proposed algorithm consists of the following six steps: (1) Preprocessing of the EEG; (2) Assessment of the EEG complexity via computation of the multiscale entropy; (3) Tensorization of each EEG recording, (4) Decomposition of the multiscale entropy tensor; (5) Selecting the component of interest; and (6) Postprocessing and clustering of the temporal signature. Each step of the algorithm will be extensively described in the next paragraphs. Finally, the metrics to assess the classification performance will be explained.

### 3.1. EEG Preprocessing

In order to avoid distortion of the EEG time series by high or low frequency artifacts, a bandpass finite impulse response filter between 1 and 40 Hz was applied on each EEG channel. Moreover, an additional notch filter at 50 Hz is used to remove any remaining powerline interference. The filters were applied in both forward and reverse directions in order to avoid phase distortion. The EEG signal is then downsampled by a factor of two to reduce the computational complexity. No advanced artifact removal or visual preselection of data has been performed.

### 3.2. Multiscale Entropy Computation

After filtering the data, the EEG signal is segmented into nonoverlapping windows of 100 s [11]. To assess the complexity of each of these multichannel EEG segments, the multiscale entropy is computed. Multiscale entropy evaluates the complexity of a signal by measuring the regularity of the signal at multiple time scales [19,20]. So, the first step in its computation is to obtain signals at different scales. The coarse-grained signal $y_{j}^{\tau }$ at scale τ is obtained by taking the average of all data points within consecutive nonoverlapping windows of length τ. So the coarse-grained time series of the signal {$x_{1},x_{2},\ldots ,x_{N}$} at scale factor τ can then be written as:

(1) $$y_{j}^{\tau }=\frac{1}{\tau }\sum_{i=(j-1)\tau +1}^{j\tau }x_{i},\;1⩽j⩽\frac{N}{\tau }.$$

This actually corresponds to a moving average operation with a window of length τ followed by a downsampling with factor τ. Hence, at scale 1 the signal is simply the original time series, while with increasing scale the coarse-grained signal length reduces progressively.

**Figure 1:**
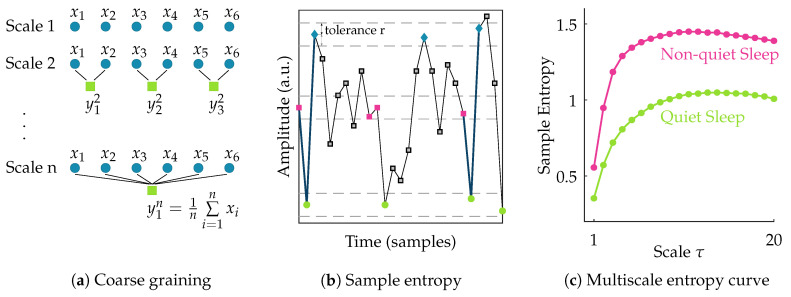
Illustration of the procedure to compute multiscale entropy (MSE). The first step (**a**) consists of coarse graining the time series into different scales; In the second step (**b**), the sample entropy of each of these coarse grained time series is computed; Finally, in (**c**), a multiscale entropy curve can be constructed by plotting the sample entropy in function of scale. The multiscale entropy curve is usually lower during quiet sleep compared to non-quiet sleep.

Following the coarse-graining of the EEG segment, the regularity of the signal at each scale τ is quantified using sample entropy. Sample entropy is a measure of the regularity or predictability of a time series. It is computed as the negative natural logarithm of the conditional probability that two sequences of *m* consecutive data points matching within a tolerance *r*, will also be similar when an additional data point is added to the sequence. For a discrete time series of length *N*, this can be expressed as [21]:

(2) $$SampEn\left(m,r,N\right)=-ln\frac{A}{B}$$

where *A* and *B* denote the number of matches of length *m* + 1 and *m* within tolerance *r*, respectively. In this study, the template length m is set equal to 2. The multiscale entropy (MSE) is computed for scales ranging from 1 to 20. The tolerance r is defined as 0.2× the standard deviation of the original time series [11,22]. Once the sample entropy is computed for each of the coarse-grained time series, a multiscale entropy (MSE) curve can be constructed. This curve shows the sample entropy in function of the scale factor τ. Hence, it reflects the regularity of the signal across multiple scales. The procedure to compute multiscale entropy of a time series is presented in Figure 1. The multiscale entropy curve of each 100 s multichannel EEG segment is computed.

### 3.3. Tensorization

The entropy values of each EEG recording are then organized in a third order tensor with modes: channels, scales and time segments. So, the multiscale entropy curves of consecutive time segments are stacked in the third mode of the tensor $X\in R^{N\times S\times T}$. Using this tensorization the structural information among the leads is preserved. In this study, the number of EEG channels N is equal to 8, the number of scales S for which the sample entropy is computed is equal to 20 and the number of time segments T depends on the length of the EEG recording. Thus, the data of each EEG recording is transformed into a third order tensor $X\in R^{8\times 20\times T}$, where each row fiber (mode-2 fiber) of the tensor represents a multiscale entropy curve from a specific EEG channel and time segment. In the remainder of this paper, this tensor will be referred to as the multiscale entropy tensor.

### 3.4. Tensor Decomposition

Throughout this paper, a scalar, vector, matrix and tensor will be denoted by lowercase letters (a), boldface lowercase letters (a), boldface capitals (A) and letters in calligraphic script (T), respectively. The outer product is denoted by ∘ and ${∥.∥}_{F}$ stands for the Frobenius norm.

The canonical polyadic decomposition (CPD) or parallel factor analysis (PARAFAC) of a rank-*R* tensor T factorizes the tensor in a sum of *R* rank-1 tensors [23]. This can be written as:

(3) $$T=\sum_{r=1}^{R}a_{r}\circ b_{r}\circ c_{r}=〚A,B,C〛$$

where the factor vectors $a_{r},b_{r}$ and $c_{r}$ are the *r*th (1⩽r⩽R) column of the factor matrices A,B and C, respectively. The factor matrices A,B and C are obtained by solving the least-squares optimization problem with objective function:

(4) $$\;\min_{A,B,C}\;\frac{1}{2}∥T-〚A,B,C〛{∥}_{F}^{2}.$$

The advantage of CPD compared to matrix factorizations is that the decomposition is unique (up to scaling and permutation ambiguity) under mild conditions [24]. Moreover, prior knowledge of the data properties can easily be taken into account by imposing constraints on the factor matrices (e.g., sparsity, smoothness, non-negativity, etc.) [15]. Because entropy values are positive, non-negativity is enforced during the decomposition of the multiscale entropy tensor. Moreover, the non-negativity constraint circumvents the occurrence of degenerate components.

**Figure 2:**
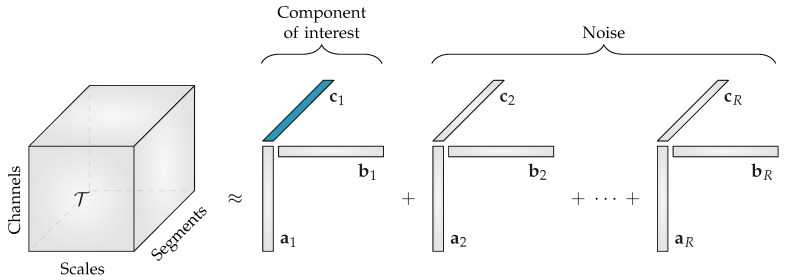
The rank-R canonical polyadic decomposition of a multiscale entropy tensor T. When the rank R is greater than 1, the component of interest related to the neonatal sleep staging has to be selected. The temporal signature of interest $c_{1}$, which is highlighted in blue, will then be used to discriminate the neonatal sleep stages. All other components will be discarded during further analysis.

The CPD of the third-order multiscale entropy tensor with rank R will result in three non-negative factor matrices: the spatial signatures will form the columns of $A\in R^{N\times R}$, the scale signatures the columns of $B\in R^{S\times R}$ and the temporal signatures the columns of $C\in R^{T\times R}$. An example of this decomposition is shown in Figure 2. The factor vectors in the first mode $a_{r}$, will show the variation over the different EEG channels, the factor vectors in the second mode $b_{r}$, contain information about the distribution over scales, while the factor vectors in the third mode $c_{r}$, will capture the variation of the EEG complexity over the different time segments.

Starting from the assumption that the EEG complexity is different depending on the sleep stage [11], we expect that one of the temporal signatures will reflect the sleep cycling. Since the goal of the algorithm is to perform automated sleep staging, only the factor vectors in the third mode, the temporal signatures, are of interest.

#### 3.4.1. Detection of Stable Solution

Due to the fact that (4) is a non-convex optimization problem, we may end up in a local minimum rather than a global minimum. Thus, different factor matrices can be obtained depending on their initialization [25]. To ensure that a reliable solution is found, the decomposition is repeated 50 times with different random initializations. Next, we want to detect and select the most occurring, stable solution among these 50 repetitions. To this end, the similarity between all possible combinations of components is assessed by means of cosine similarity. In this study we assume that each component should match in all its modes, so the triple cosine product or congruence is computed. To assess the similarity of two rank 1 tensors X=k∘l∘m and Y=p∘q∘r this can be written as [26,27,28]:

(5) $$\mathrm{cong}\left(X,Y\right)=\cos\left(k,p\right)\cos\left(l,q\right)\cos\left(m,r\right)=\frac{k^{T}p}{∥k∥∥p∥}\frac{l^{T}q}{∥l∥∥q∥}\frac{m^{T}r}{∥m∥∥r∥}.$$

The R components of each repetition are sorted based on (5) to account for the permutation indeterminacy of the decomposition. Subsequently, the similarity between the corresponding components of different repetitions is investigated. On the assumption that all R components should match, the congruence of the R components is multiplied. This can be organized in a symmetric similarity matrix $S\in R^{it\times it}$, where *it* represents the number of iterations (in this case 50). So, $s_{ij}$ represents how similar the factor matrices of the *i*th and *j*th iteration are. Finally, the solution of the iteration with the highest similarity to the other repetitions is selected as the stable and reproducible solution.

#### 3.4.2. Number of Components

One of the principal challenges when applying tensor decompositions is selecting an appropriate number of components for the problem at hand [29]. A multitude of strategies to define the rank have been proposed. First of all, we investigated the multilinear singular spectrum in order to get an initial estimate of the rank. Then, we performed the decomposition for rank R going from 1 to 5. To compare the different ranks, we investigated the core consistency diagnostic (CORCONDIA), which assesses how close the core tensor is to being superdiagonal. In addition, a diagnostic based on the relative reduction of the fitting error for an increase of the rank, called the difference of fit (DIFFIT) [30], was computed to assist in determining the appropriate number of components.

### 3.5. Selection of the Component of Interest

When decomposing the multiscale entropy tensor, we expect that one component will reflect the sleep staging of the neonate. However, if the number of components R is greater than one, an automatic selection of the component of interest is required. So, the goal is to find the temporal signature of the component of interest. In Figure 2 this is the temporal signature of the first component, ($c_{1}$) which is marked in blue.

We expect that the component related to sleep staging will have a more regular, cyclic pattern compared to the other (noise) components. Therefore, the autocorrelation functions of the temporal signatures are computed. Since the temporal signature reflecting the sleep staging has a stronger correlation in time, we expect that its area under the absolute value of the autocorrelation function (ACF) will be larger [31,32]. Therefore, the component whose area is the largest will be selected as the component of interest and used for further processing.

**Figure 3:**
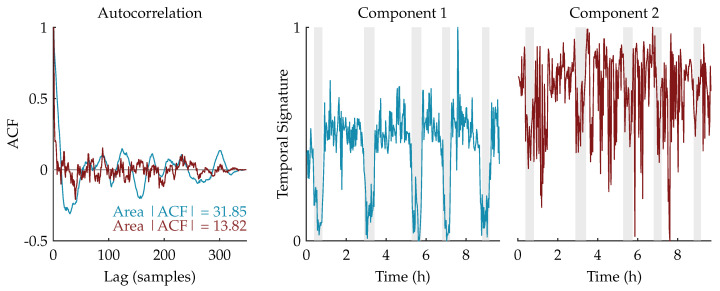
On the left, the autocorrelations of the temporal signatures of a rank-2 canonical polyadic decomposition (CPD) of a multiscale entropy tensor are shown. The area under the absolute value of the autocorrelation is equal to 31.85 and 13.82 for the first component (in blue) and the second component (in red), respectively. Hence, the first component will be selected as the component of interest. On the right, the corresponding temporal signatures are plotted with the annotated quiet sleep periods highlighted in light grey. The temporal signature of the first component shows a clear reduction during quiet sleep, while the second component is not correlated to the sleep cycling.

Figure 3 shows an example of this procedure for a rank-2 CPD of a multiscale entropy tensor. The autocorrelation of the two temporal signatures is plotted on the left. The area under the absolute value of the autocorrelation is equal to 31.85 and 13.82 for the temporal signature of the first (blue) and second (red) component, respectively. Therefore, the first component will be selected as the component of interest. The temporal signatures of the two components are plotted on the right with highlighted periods of quiet sleep in light grey. From these graphs we can see that the first component is indeed reduced during quiet sleep segments compared to non-quiet sleep segments, whereas the second component is not related to the sleep stages.

### 3.6. Postprocessing and Clustering

Once the non-negative polyadic decomposition of the multiscale entropy tensor is performed, the temporal signature of the selected component is used to define the neonate’s sleep stage. This temporal signature shows a (slow) cyclic pattern reflecting the sleep staging of the infant. However, there are high frequency oscillations superimposed on this pattern, which could lead to incorrect sleep stage identification. Therefore, a postprocessing step consisting of smoothing the temporal signature using a moving average filter with length L equal to 5 is applied. This moving average filter is applied in both directions (to avoid phase distortion), so actually a weighted moving average filter is used with triangular shape and length 2 × L. This smoothing operation accounts for the fact that a sleep stage does not change instantly. More specifically, sleep periods were only visually labeled as quiet sleep if 3 consecutive minutes or 3 out 4 min were clinically detected as quiet sleep.

In order to divide the data then into two distinct clusters, k-means clustering (k = 2) is performed using the smoothed temporal loading. So, each data point of the smoothed temporal signature of interest $c\in R^{T\times 1}$ will be assigned to one of the two clusters. Thus, the result of the clustering is a vector of length T containing a cluster label for each of the 100 s EEG segments. Since k-means clustering is heavily dependent on its initialization, k-means clustering is repeated 100 times with different initial cluster centroids and the clustering with the lowest sum of within-cluster point-to-centroid distances is selected. The cluster with the lowest EEG complexity will be assigned the label of quiet sleep, while the other cluster is labeled as non-quiet sleep [11,33].

**Figure 4:**
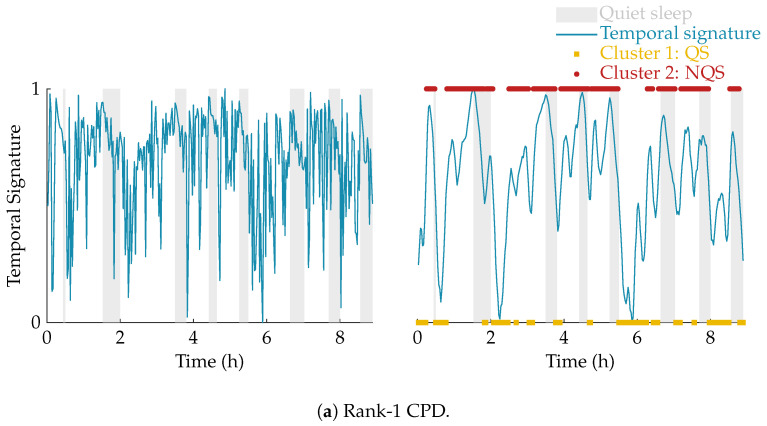

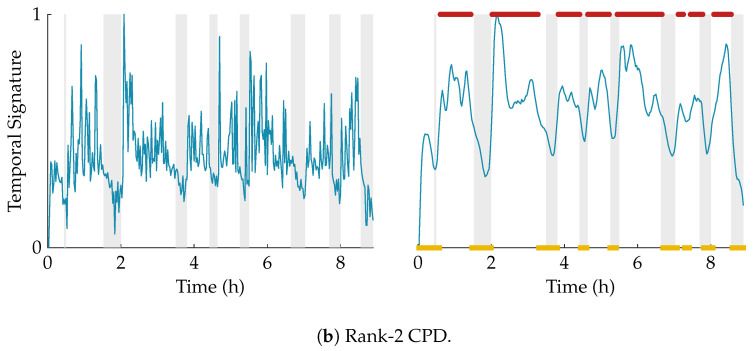
Illustration of the postprocessing and clustering of the temporal signature for a rank-1 (**a**) and rank-2 (**b**) decomposition of the multiscale entropy tensor (PMA = 40 weeks 5 days). On the left half of the figure, the temporal signature of interest is plotted in blue. The right half of the figure shows the smoothed temporal signature after applying the weighted moving average filter. Moreover, the quiet sleep and non-quiet sleep segments estimated by the algorithm are marked by the yellow squares and red dots, respectively. The clinically labelled quiet sleep periods are highlighted in light grey. The rank-1 CPD does not give a good indication of the sleep stages, while the (smoothed) temporal signature of the rank-2 CPD is clearly reduced during the quiet sleep periods.

The effect of the smoothing and the result of the clustering is illustrated in Figure 4. In this example, the multiscale entropy tensor of an EEG recording is decomposed for rank R equal to 1 and 2. The raw temporal signatures of the rank-1 and rank-2 decomposition (the only temporal signature of interest) are shown on the left, while their smoothed versions are plotted on the right. The smoothing clearly removes the unwanted variations. The sleep labels obtained by the algorithm based on k-means clustering are marked by yellow squares (cluster 1 corresponding to quiet sleep) and red dots (cluster 2 corresponding to non-quiet sleep). The rank-1 CPD does not correlate well with the clinical annotations of quiet sleep highlighted in light grey, while the smoothed temporal signature of the rank-2 CPD is clearly reduced during quiet sleep.

### 3.7. Classification Performance

The performance of the algorithm is evaluated using the annotations by expert clinicians. The sensitivity, specificity and accuracy will be reported. Moreover, to investigate the performance without the k-means clustering, receiver operating characteristic (ROC) curves are constructed based on the smoothed temporal signatures and the clinical labels. The area under the ROC curve (AUC) will be reported. Finally, Cohen’s kappa will be presented as well.

### 3.8. Statistical Analysis

Statistical analysis is performed to gain insight in the choice of the parameters. A statistical test is used to investigate whether the area under the absolute value of the autocorrelation function is a good feature to detect the component of interest. In order to do this, the agreement between each of the R temporal signatures obtained from a rank-R CPD and the clinical sleep labels are evaluated using the Kappa score. The temporal signatures are then sorted in descending order based on their Kappa statistic. As a result, the first component is the most related to sleep staging and should have the largest area under the absolute value of its autocorrelation function (ACF). A statistical test is then performed to assess whether the area under the ACF of the component of interest is significantly different from the other components. In addition, a statistical test is carried out to investigate the influence of the rank R on the performance. More specifically, we compared Cohen’s Kappa between models with different values of the rank R. In all analyses, the Shapiro-Wilk test is used to test for normality. If the data is normally distributed, one-way ANOVA is used, otherwise a Kruskal-Wallis test is performed. The significance level is always set equal to 0.05.

## 4. Results

**Figure 5:**
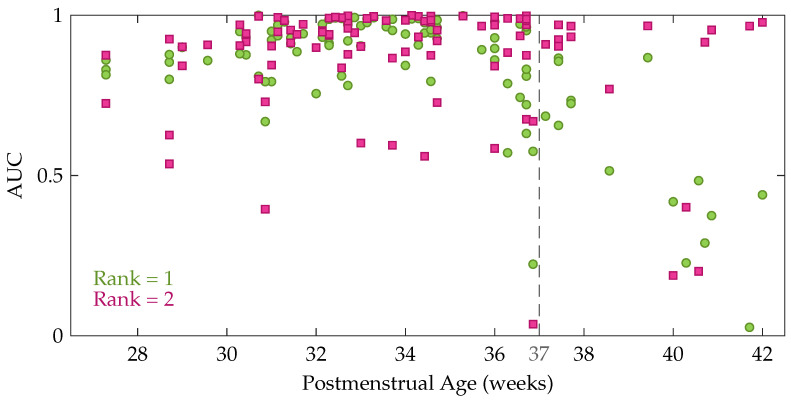
The area under the ROC curve as a function of the postmenstrual age at the moment of the recording. Each green circle shows the performance of one of the EEG recordings for a rank-1 CPD, whereas the pink squares represent the performance for a rank-2 CPD.

Comparison of the factor matrices obtained using the rank indicated by CORCONDIA or DIFFIT with the ones for other ranks revealed that the diagnostics were often not suitable to define the appropriate number of components in this application. Therefore, the rank has not been fixed for each recording, instead the results are reported for different values of the rank R. Table 1 presents the mean and standard deviation of the performance measures. The first five rows show the performance for a fixed rank R for all recordings with R going from 1 to 5. From these data, we can see that the average performance is slightly higher for rank 2 compared to rank 1 (higher AUC and kappa). Moreover, Table 1 also shows that the performance decreases gradually for an increasing rank beyond 2. However, a Kruskal-Wallis test with multiple comparisons revealed that this performance difference between the rank-1 and rank-2 model is not significant, while Cohen’s kappa of the rank-1 and rank-2 model are both significantly better compared to the rank-5 model. In order to get more insight in the performance difference between a rank-1 and rank-2 CPD, the performance of each recording is plotted as a function of the postmenstrual age in Figure 5. In this figure each green circle and pink square represents the area under the ROC curve of an EEG recording for a rank-1 and rank-2 decomposition, respectively. The dashed line indicates 37 weeks postmenstrual age. The most interesting aspect of this graph is that when a rank-1 decomposition is used, a high performance is only obtained up to around 36 to 37 weeks PMA. From that age onwards a clear drop in the AUC can be observed in Figure 5. The rank-2 CPD on the other hand, has a high performance for most recordings at term equivalent age, but has a lower performance for some measurements recorded at a younger age.

Based on these findings we decided to also report the results for an age-dependent rank, where the rank is chosen equal to 1 for preterm recordings and equal to 2 for EEGs recorded from 37 weeks PMA onwards. The performance of this approach is set out in the sixth row of Table 1. It is clear that the average performance of using an age-dependent rank is better compared to a fixed rank for all recordings, however this performance difference is not significant.

Finally, the last two rows of the table show the classification performance when the optimal rank (between 1 and 5) for each recording was selected based on Cohen’s kappa computed with the ground truth annotations. Specifically, the last row corresponds to the highest attainable performance (optimal rank and best component), while in the penultimate row the optimal rank was used but the component of interest was automatically selected using the strategy based on the autocorrelation explained above. As a consequence, the difference between these two rows is caused by failures of the automatic component selection procedure.

**Figure 6:**
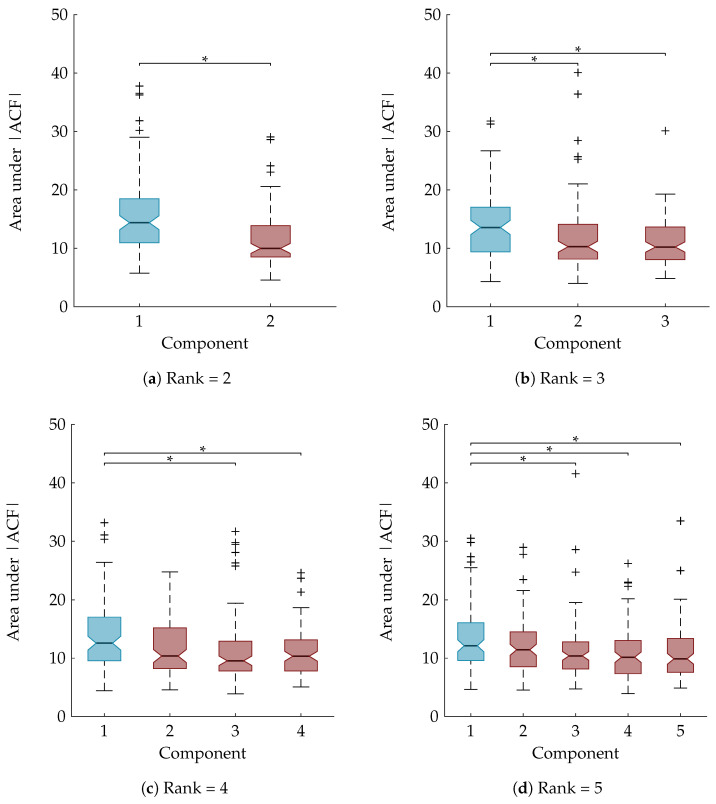
Boxplots of the area under absolute value of autocorrelation of the temporal signatures after sorting them in descending order according to their Kappa score. The component with the largest Kappa score and therefore most related to sleep staging is the first one and is marked in blue, while the other (noise) components are marked in red. “*” indicates a statistically significant difference with *p* < 0.05. For the rank-2 CPD (**a**) and the rank-3 CPD (**b**) there is a significant difference between the area under the ACF of the sleep staging component and the noise component(s); For a rank-4 (**c**) and rank-5 CPD (**d**) the component most related to sleep staging is significantly different from all other components except one.

As explained in Section 3.8, a statistical analysis is performed to examine whether the area under the ACF is a suitable feature to rely on for the automatic component selection. The boxplots in Figure 6 show the area under the absolute value of the ACF after sorting the temporal signatures based on their agreement with the clinical annotations. The component of interest, with the largest Kappa score, is the first component and is marked in blue. A Mann-Whitney U test and Kruskal-Wallis test with multiple comparisons determined that the first component is significantly different from all other components for the rank-2 and rank-3 CPD, respectively (Figure 6a,b). For the rank-4 and rank-5 CPD the area under the ACF of the sleep staging component is significantly different compared to all other components except the second one. This is also indicated on the boxplots in Figure 6c,d.

## 5. Discussion

This study aimed to discriminate quiet sleep (QS) from non-quiet sleep (NQS) using the nonlinear dynamics of the EEG signal in a data-driven way. The proposed algorithm relies on the fact that the EEG complexity is different depending on the sleep state. In order to quantify the complexity of the EEG signal, multiscale entropy is computed for consecutive segments of a multichannel EEG recording. These multiscale entropy values are then used to construct a third-order tensor with modes channels, scales and segments. Subsequently, a rank-R nonnegative CPD of the multiscale entropy tensor is computed. The temporal signature of interest is detected based on the area under the absolute value of its autocorrelation function. After smoothing, this temporal signature shows a cyclic pattern reflecting the neonatal sleep staging. Clustering is then performed to discriminate quiet sleep from non-quiet sleep.

The performance of the algorithm is reported for different ranks. The average Kappa is equal to 0.47 and 0.49, for a rank-1 and rank-2 CPD, respectively. However, if a rank-1 decomposition is used for EEGs recorded at a postmenstrual age below 37 weeks and a rank-2 decomposition is used for EEGs recorded at an older age, a Kappa of 0.53 is obtained. This indicates that a higher rank is preferred for neonates at term equivalent age, while rank 1 is suitable for most of the preterm recordings. The fact that for some recordings rank 1 is not sufficient, is demonstrated by the example in Figure 4. An even higher performance could be obtained if the optimal rank is used and the component of interest is detected correctly. However, as can be seen from Table 1, the performance when the optimal rank is used and the component of interest is selected automatically is slightly lower compared to an age-dependent rank. This reduction in performance is due to the fact that the procedure to select the component of interest is more likely to fail for a higher number of components. From the data presented in Table 1 we can see that the performance gradually decreases if a rank higher than two is used. This finding suggests that for the majority of the recordings in the current database a lower rank is more appropriate. Nevertheless, this performance reduction can also be partly attributed to the more difficult component selection for higher ranks.

There are various possible explanations for the need for a higher rank for recordings at term-equivalent age. First of all, it is likely that this is due to the increased amount of artifacts in near term EEG recordings. It is expected that an older neonate will move more, which could lead to more severe distortions and lower quality of the EEG signal. The higher performance for higher ranks in noisy preterm EEG recordings confirms this reasoning. Secondly, the emergence of four distinct sleep states around 36 weeks PMA might also play a role in this phenomenon [4].

The performance of the described algorithm is lower compared to state-of-the-art algorithms [13,14,34]. However, there are multiple advantages of the proposed method. The major advantage of the algorithm is that it is data-driven and unsupervised. As a consequence, the method can be easily applied on a completely new dataset and used in new centers, where there is little expertise about EEG sleep labelling. Moreover, in this study the algorithm is assessed on eight channel EEG recordings measured using the restricted 10–20 system for neonates. However, the same approach can also be applied on datasets with fewer or more electrodes without adjustments. Finally, the tensor decomposition can be updated in an efficient way whenever a new batch of EEG data is available [35]. This allows real time tracking of neonatal sleep states.

The main weakness of this study is the lack of a method to define the rank for each new EEG recording automatically in a data-driven way. This is a general issue in tensor applications, therefore it is suggested to combine different tools to asses the rank [36,37]. We tested several diagnostics, but none of them resulted in a reliable estimate of the number of components. More research is needed to determine the optimal number of components for this specific application. An additional drawback of the proposed algorithm is the procedure for selecting the component of interest. The temporal signature of interest does not always have the largest area under the absolute value of the autocorrelation function. For that reason more advanced strategies for component selection should be investigated in future studies. Another limitation of the study is that artifacts commonly lead to misclassification of the segment. More specifically, EEG segments distorted by artifacts are often classified as quiet sleep because they have a reduced EEG complexity. The cause of this is two-fold. First of all, artifacts are more predictable and less complex compared to noise-free EEG [38]. Secondly, a high amplitude artifact will drastically increase the standard deviation of the segment, resulting in a much higher tolerance *r* and thus more template matches and consequently lower entropy. Detecting artifacts as quiet sleep periods will lead to a reduced performance, since the majority of the (motion) artifacts occur during non-quiet sleep. The smoothing operation is implemented to reduce the influence of artifacts and can deal with short duration artifacts (sudden drop in EEG complexity). Yet, the postprocessing cannot avoid false detections caused by longer artifacts (on multiple channels). Further research might explore incorporating information about the noise in the algorithm to make it more robust to artifacts. Besides, an estimation of the amount of noise could be used to get an initial estimate of the proper number of components. Notwithstanding these limitations, the study suggests that a decomposition of a multiscale entropy tensor can be used to discriminate neonatal sleep stages.

This study is an exploratory analysis on the use of tensor decompositions for sleep stage identification in preterm infants. Only the differences in complexity between different sleep stages are used. However, in future work the temporal signature could be combined with other discriminating features (e.g., spectral edge frequency, power in specific EEG frequency bands, etc.) to boost the performance. Moreover, as explained before, the heart rate variability, respiration and body movements of the infant are also dependent on the sleep state. Hence, in future research the combination of these complementary modalities can be studied. Finally, other tensorization techniques can be examined in future investigations.

## 6. Conclusions

This study confirmed that the complexity of brain dynamics exhibits fundamental differences between vigilance states in preterm newborns. The EEG complexity is significantly lower during quiet sleep compared to non-quiet sleep. This property is exploited to develop an unsupervised algorithm that can detect quiet sleep in preterm infants based on the data-driven factorization of the multiscale entropy tensor.

## Figures and Tables

**Table 1:** The classification performance of the proposed tensor-based method for different values of the rank R. The mean (standard deviation) of the sensitivity, specificity, accuracy, area under the ROC curve (AUC) and Cohen’s kappa are presented.

		Sensitivity	Specificity	Accuracy	AUC	Kappa
*Fixed rank*						
	R = 1	0.73(0.29)	0.79(0.16)	0.78(0.15)	0.84(0.19)	0.47(0.34)
	R = 2	0.82(0.27)	0.74(0.23)	0.76(0.18)	0.87(0.19)	0.49(0.33)
	R = 3	0.74 (0.34)	0.71(0.2)	0.72(0.17)	0.80(0.24)	0.38(0.36)
	R = 4	0.75(0.33)	0.67(0.22)	0.70(0.18)	0.80(0.24)	0.35(0.36)
	R = 5	0.73(0.35)	0.64(0.24)	0.67(0.21)	0.75(0.27)	0.31(0.38)
*Age-dependent rank*						
	Preterm: R = 1, term: R = 2	0.80(0.23)	0.79(0.17)	0.79(0.14)	0.87(0.16)	0.53(0.28)
*Optimal rank*						
	Automatic component selection	0.76(0.32)	0.78(0.18)	0.79(0.17)	0.85(0.21)	0.50(0.38)
	Optimal component	0.86(0.32)	0.81(0.18)	0.82(0.17)	0.91(0.21)	0.60(0.38)

## Abbreviations

The following abbreviations are used in this manuscript:

ACF	Autocorrelation function
AUC	Area under the curve
CORCONDIA	Core consistency diagnostic
CPD	Canonical polyadic decomposition
DIFFIT	Difference of fit
EEG	Electroencephalogram
FIR	Finite impulse response
MSE	Multiscale entropy
NICU	Neonatal intensive care unit
NQS	Non-quiet sleep
PMA	Postmenstrual age
QS	Quiet sleep
ROC	Receiver operating characteristic
